# Togolese Doctors’ Awareness, Perceptions, and Practices of Telemedicine: A National Cross‐Sectional Study

**DOI:** 10.1155/jotm/4658443

**Published:** 2026-01-20

**Authors:** Kokou Adambounou, Raymondo Sêdzro Kuto, Akoété Beleave Kouevidjin, Alexis Agbodjan

**Affiliations:** ^1^ Telemedicine Unit, Campus Teaching Hospital, University of Lomé, Lomé, Togo, univ-lome.tg; ^2^ Medical Imaging Department, Campus Teaching Hospital, University of Lomé, Lomé, Togo, univ-lome.tg

**Keywords:** digital health, physicians, telehealth, telemedicine, Togo

## Abstract

**Objective:**

To assess the awareness, perceptions, and practices of telemedicine among Togolese doctors.

**Material and Methods:**

Descriptive cross‐sectional study was conducted from January to March 2021 on Togolese doctors residing and practicing in Togo. A structured questionnaire which included 32 questions (closed‐ended and multiple‐choice items) was developed. Data were collected electronically using a Google Form sent by e‐mail and WhatsApp to the doctors.

**Results:**

Of the 136 doctors surveyed, 83.8% were specialists and 33.8% were university hospital doctors. The internet connection at their place of practice was considered to be mediocre by 44.1% and acceptable by 40.4%. Awareness of teleconsultation (92.7%), tele‐expertise (61%), and medical teleassistance (52.9%) was reported. A minority were aware of telemonitoring (43.4%) and medical regulation (14%). Around two‐thirds of doctors were aware of the need for patient consent prior to telemedicine (65.4%). Telemedicine was perceived as a necessity in 67.7% of cases. Telemedicine was practiced by 68.4% and this was teleconsultation in 57.4% and tele‐expertise in 21.3%. Majority (90%) used WhatsApp to consult and prescribe a prescription remotely. Around 7% and 4% used Facebook for teleconsultation and tele‐expertise, respectively. It was opined that medical imaging (74.3%), general medicine (63.2%), and dermatology (56.6%) were the areas in which telemedicine should be further developed.

**Conclusion:**

The level of awareness of telemedicine among Togolese doctors was suboptimal. Good practices were not always adopted and perceptions were relatively encouraging. Ongoing training and awareness raising on telemedicine good practices could be necessary for its efficient and safe use in Togo.

## 1. Introduction

Telemedicine is the practice of medicine using interactive data communication techniques [1]. It is a form of medicine that is adapted to its environment, to advances in science, and to societies that have become familiar with new information and communication technologies [2].

Telemedicine consists of 5 main procedures: teleconsultation, tele‐expertise, telemonitoring, teleassistance, and medical regulation. It has provided solutions to the challenges posed by socioeconomic changes in healthcare systems in the 21^st^ century, such as increased demands for healthcare, aging populations, increased mobility of citizens, the need to manage large amounts of information [2]. In addition, global competitiveness, improved healthcare delivery, all in a context of globalization, limited budgets, and spending restrictions have benefited from telemedicine [2]. In recent years, telemedicine has undergone a significant evolution, especially since the onset of COVID‐19 pandemic during which containment measures and restrictions on people’s movement limited physical contact with healthcare professionals.

Unlike in Europe or the USA, where telemedicine is booming and frequently used in day‐to‐day patient care [3], most telemedicine initiatives in Africa, particularly sub‐Saharan Africa, are limited to pilot projects [4]. Analysis of these projects shows that a number of conditions are essential for the sustainable implementation of telemedicine in Africa particularly the role of healthcare professionals [5, 6].

In Togo, the first telemedicine initiatives began in the 2010s with the low‐cost telemedicine platform piloted between the Tours University Hospital in France, the Campus University Hospital in Lomé, and the Tsévié Regional Hospital in Togo [7, 8]. This low‐cost platform made it possible to carry out not only teleimaging procedures, in particular tele‐echography [9, 10], but also teleconsultation, teledermatology, and teletraining. However, the development of a telemedicine platform depends on the support of doctors. This in turn depends on their awareness and perceptions of telemedicine. However, no study has been conducted on this specific subject in Togo. It was with this in mind that we undertook this study. The aim of which was to assess the awareness, perceptions, and practices of telemedicine among Togolese doctors.

## 2. Materials and Methods

### 2.1. Study Design and Setting

This descriptive cross‐sectional questionnaire‐based study was conducted from January to March 2021. Togo is located in West Africa, with a surface area of 56,600 km^2^ and an estimated population of 8.5 million in 2020 [11]. According to a report by Togo’s postal and telecommunications regulatory authority, the proportion of the population connected to fixed and mobile broadband internet was 61.7% in 2019, with the number of fixed broadband internet subscribers rising steadily [12]. In Togo, six operators are present on the electronic communications market and offer fixed telephone, mobile telephone, fixed internet (FTTH, ADSL, and WiMAX technologies), and mobile internet services (2G, 3G, and 4G and has a license to experiment with 5G.). The penetration rate for fixed and mobile Internet, all technologies combined, was 61.7% in 2019. Fixed and mobile broadband penetration reached 44% in 2019 [12].

### 2.2. Study Population

Our study included all Togolese doctors residing and practicing in Togo, who had given their informed consent to participate in the study. Togolese doctors not residing or practicing in Togo, those who had not given informed consent to participate in the study, foreign doctors residing and practicing in Togo, and veterinary surgeons were not included in this study.

### 2.3. Sampling Procedures

An exhaustive sampling strategy was used. According to the 2020 official register of the national order of physicians, 241 medical doctors were listed and up to date with registration requirements. All were invited to participate.

### 2.4. Data Collection Instrument and Variables

A structured questionnaire was developed specifically for this study, based on the variables of interest and pretested prior to administration. It included 32 questions (closed‐ended and multiple‐choice items). Collected variables comprised the following:

 Sociodemographic characteristics (gender, qualifications, specialty, years of experience, country of general medicine training, country of specialization, postdoctoral training, practice type, and location).
 Awareness of telemedicine (objective knowledge of telemedicine concepts, definitions, and technological, organizational, legal, and regulatory aspects).
 Perceptions of telemedicine (subjective views regarding its value, feasibility, and relevance in Togo).

The English version of the questionnaire was provided as supporting information in the original study.

### 2.5. Data Collection Procedures

Data were collected electronically via a Google Forms survey. The form link was sent by e‐mail and WhatsApp to the doctors mainly via various doctors’ WhatsApp group platforms available during the study period. The WhatsApp group platforms targeted were in particular those created by the various doctors’ associations in Togo and those of the different hospitals in Togo. The URL link was also emailed individually to some doctors.

Before accessing the questionnaire, all participants were informed of the study objectives, the voluntary nature of participation, and their right to withdraw at any stage without consequences. This information was displayed both on the Google Forms interface and in email invitations. To protect participants’ privacy and confidentiality, the Google Forms questionnaire was configured to collect no personal identifiers, such as names, phone numbers, IP addresses, or email accounts. Responses were stored in an encrypted Google Workspace environment accessible only to the principal investigators through password‐protected accounts.

### 2.6. Data Processing and Analysis

Our collected data were exported in CSV format. Microsoft Excel 2019 was used to check and format the data. Stata version 15.1 was used to analyze the data. Quantitative data were presented in the form of absolute and relative frequencies (percentages). Graphs were produced in Microsoft Excel 2019. Chi‐square and Fisher’s exact tests were used to compare percentages. Differences were considered statistically significant for a *p*‐value of less than 0.05.

### 2.7. Ethical Considerations

The study received authorization from the Ministry of Health. The questionnaire was anonymous, ensuring confidentiality and data protection for all participants. Informed consent was obtained electronically.

## 3. Results

### 3.1. Sociodemographic Characteristics

Our sample consisted of 136 doctors representing 56.4% of the number of doctors registered and up to date with the national order of doctors in Togo during the study, 116 (85.3%) of whom were male. Majority of the doctors were aged between 26 and 35, had less than 10 years’ professional experience, trained and specialized in Togo (Table 1). Table 1 also shows that the most common specialties were surgery, paediatrics, radiology, gynecology, and ophthalmology.

**Table 1:** Distribution of sociodemographic characteristics of the doctors surveyed.

	*n*	%
Sex		
Male	116	85.3
Female	20	14.7
Age range		
26–35 years old	70	51.47
36–45 years old	54	39.7
46–55 years old	9	6.62
56–65 years old	1	0.74
> 65 years old	2	1.47
Professional experience		
< 5 years	64	47.06
6–10 years	35	35
11–15 years	21	15.44
> 15 years	16	11.76
Country of training		
Togo	117	86.03
Foreign	19	13.97
Country of specialization		
Togo	79	69.29
Foreign	35	30.71
Specialties		
General medicine	22	16.18
Surgery	16	11.76
Paediatrics	15	11.03
Radiology	15	11.03
Gynecology	14	10.29
Ophthalmology	10	7.35
Occupational medicine	5	3.68
Nephrology	5	3.68
Otorhinolaryngology	5	3.68
Internal medicine	4	2.94
Intensive care anesthesia	3	2.21
Dermatology	3	2.21
Psychiatry	3	2.21
Public health	3	2.21
Urology	3	2.21
Cardiology	2	1.47
Pneumology	2	1.47
Others	6	4.41

*Note:* Others (anatomopathology, biology, forensic medicine, neurology, and rheumatology).

Eighty‐seven doctors (64%) worked in the city of Lomé and 49 doctors (36%) worked in the interior of the country (outside Lomé, the capital). Ninety doctors (66.2%) were hospital doctors and 46 doctors (33.8%) were university hospital doctors. Twenty‐two doctors (16.2%) were general practitioners and 114 doctors (78%) were specialists. Twenty‐nine doctors (21.3%) practiced in private practice, 77 doctors (56.6%) practiced in public practice, and 30 doctors (22.1%) practiced in both private and public practice.

The speed of the internet connection in the place of work was described as mediocre by 60 doctors (44.1%). It was considered acceptable by 55 doctors (40.4%) and good by 9 doctors (6.6%). Eight doctors (5.9%) noted the absence of an internet connection in their departments.

### 3.2. Doctors’ Awareness of Telemedicine

All the doctors who took part in the survey said they had heard of telemedicine at least once. Ten doctors (7.4%) were not aware of any telemedicine procedures, 126 doctors (92.7%) were aware of the procedure of teleconsultation, 83 doctors (61%) were aware of tele‐expertise. Seventy‐two doctors (52.9%) were aware of medical teleassistance. Fifty‐nine doctors (43.4%) were aware of medical telemonitoring; 19 doctors (14%) were aware of medical regulation. Greater awareness of tele‐expertise was found among doctors who had completed a postgraduate training course in Europe, with a statistically significant difference, and awareness of teleassistance was higher among doctors with extensive professional experience and postgraduate training in Europe and specialist doctors (*p* < 0.05) (Table 2).

**Table 2:** Factors associated with doctors’ awareness of teleconsultation, tele‐expertise, telemonitoring, and teleassistance.

	Teleconsultation			Tele‐expertise			Telemonitoring			Teleassistance		
	*n*	%	*p*	*n*	%	*p*	*n*	%	*p*	*n*	%	*p*
Age			0.99			0.99			0.127			0.376
< 45 years old (*n* = 124)	115	92.74		76	61.29		51	41.13		64	51.61	
≥ 45 years old (*n* = 12)	11	91.67		7	58.33		8	66.67		8	66.67	
Professional experience			0.925			0.847			0.345			0.012
< 5 years (*n* = 64)	58	90.63		40	62.5		24	37.5		28	43.75	
6–10 years (*n* = 35)	33	94.29		20	57.14		16	45.71		17	48.57	
11–15 years (*n* = 21)	20	95.24		12	57.14		9	42.86		13	61.9	
> 15 years (*n* = 16)	15	93.75		11	68.75		10	62.5		14	87.5	
Place of work			0.99			0.47			0.99			0.99
Lomé (*n* = 87)	80	91.95		51	58.62		38	43.68		46	52.87	
Outside Lomé (*n* = 49)	46	93.88		32	65.31		21	42.86		26	53.06	
Postdoctoral training in Europe			0.98			0.006			0.15			0.002
No training (*n* = 88)	79	89.77		46	52.27		34	38.64		38	43.18	
Training (*n* = 48)	47	97.92		37	77.08		25	52.08		34	70.83	
Specialty			0.056			0.817			0.252			0.037
General practitioner (*n* = 22)	18	81.82		14	63.64		7	31.82		7	31.82	
Specialist (*n* = 114)	108	94.74		69	60.53		52	45.61		65	57.02	
Mode of practice			0.99			0.193			0.855			0.99
Hospital practicians (*n* = 90)	83	92.22		51	56.67		40	44.44		48	53.33	
University hospital (*n* = 46)	43	93.48		32	69.57		19	41.30		24	52.17	
Type of exercise			0.825			0.27			0.354			0.654
Private (*n* = 29)	26	89.66		16	55.17		10	34.48		14	48.28	
Public (*n* = 77)	72	93.51		45	58.44		33	42.86		40	51.95	
Private and public (*n* = 30)	28	93.33		22	73.33		16	53.33		18	60	

WhatsApp, telephone calls, and e‐mail were mentioned by 41 (30.2%), 28 (20.6%), and 10 (7.4%) doctors, respectively, as the main means of telemedicine known to them.

One hundred and sixteen doctors (85.3%) were aware that telemedicine is useful in everyday medical practice. Ninety‐seven doctors (71.3%) had already heard of a telemedicine activity or project in Togo. Eighty‐nine doctors (65.4%) were aware that it was important to have the patient’s consent before collecting and sharing information with other colleagues. The awareness of the usefulness of telemedicine in daily medical practice was more widely expressed by doctors practicing within the country (outside Lomé, the capital), with a statistically significant difference (*p* < 0.05); awareness of a telemedicine activity or project was more widely expressed by doctors who had completed a postgraduate training course in Europe, with a statistically significant difference (*p* < 0.05) (Table 3). Table 3 also shows that awareness of the importance of obtaining patient consent before collecting and sharing information with other colleagues was significantly higher among doctors practicing outside the capital (*p* < 0.05).

**Table 3:** Factors associated with doctors’ awareness of the usefulness of telemedicine in daily medical practice, of a telemedicine activity or project in Togo, and of the need to have the patient’s consent before sharing their information.

	The usefulness of telemedicine in everyday medical practice			Knowledge of a telemedicine activity or project in Togo			Knowledge of the need for patient consent before sharing information		
	*n*	%	*p*	*n*	%	*p*	*n*	%	*p*
Age			0.99			0.99			0.127
< 45 years old (*n* = 124)	115	92.74		76	61.29		51	41.13	
≥ 45 years old (*n* = 12)	11	91.67		7	58.33		8	66.67	
Professional experience			0.925			0.847			0.345
< 5 years (*n* = 64)	58	90.63		40	62.5		24	37.5	
6–10 years (*n* = 35)	33	94.29		20	57.14		16	45.71	
11–15 years (*n* = 21)	20	95.24		12	57.14		9	42.86	
> 15 years (*n* = 16)	15	93.75		11	68.75		10	62.5	
Place of work			0.99			0.47			0.99
Lomé (*n* = 87)	80	91.95		51	58.62		38	43.68	
Outside Lomé (*n* = 49)	46	93.88		32	65.31		21	42.86	
Postdoctoral training in Europe			0.98			0.006			0.15
No training (*n* = 88)	79	89.77		46	52.27		34	38.64	
Training (*n* = 48)	47	97.92		37	77.08		25	52.08	
Specialty			0.056			0.817			0.252
General practitioner (*n* = 22)	18	81.82		14	63.64		7	31.82	
Specialist (*n* = 114)	108	94.74		69	60.53		52	45.61	
Mode of practice			0.99			0.193			0.855
Hospital practicians (*n* = 90)	83	92.22		51	56.67		40	44.44	
University hospital (*n* = 46)	43	93.48		32	69.57		19	41.3	
Type of exercise			0.825			0.27			0.354
Private (*n* = 29)	26	89.66		16	55.17		10	34.48	
Public (*n* = 77)	72	93.51		45	58.44		33	42.86	
Private and public (*n* = 30)	28	93.33		22	73.33		16	53.33	

Seventy‐six doctors (55.9%) were aware of training methods for telemedicine practice. E‐learning was cited by 58 doctors (42.6%). Videoconferencing was cited by 49 doctors (36.1%). The interuniversity diploma was cited by 48 doctors (35.3%). Telemedicine journals were cited by 37 doctors (27.2%).

The main benefits of carrying out telemedicine procedures were the closer links with a hospital structure mentioned by 41 doctors (30.2%), the fight against medical desertification in rural areas by 36 doctors (26.5%), and the benefits for patients by 33 doctors (24.3%). The main obstacles to the use of telemedicine were organizational obstacles in 105 (77.2%), economic obstacles in 99 (72.8%), and medicolegal aspects in 71 (52.2%).

### 3.3. Perceptions and Practices of Doctors in Relation to Telemedicine in Togo

#### 3.3.1. Perceptions of Doctors in Relation to Telemedicine in Togo

Ninety‐three doctors (68.4%) saw telemedicine as the future of medicine. Ninety‐two doctors (67.7%) considered telemedicine to be a necessity. Four doctors (4.4%) considered telemedicine to be a matter for specialists, and 3 doctors (2.2%) did not feel concerned.

**Figure 1:**
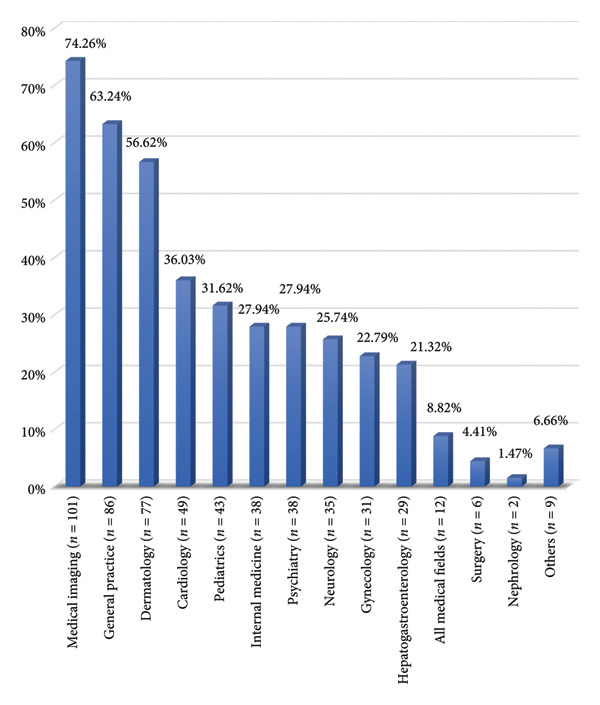
Breakdown of areas (specialties) which doctors believe should benefit from the development of telemedicine.

Medical imaging followed by general practice and dermatology were the medical fields most likely to benefit from the development of telemedicine according to the doctors (Figure 1). The ideal places for telemedicine practice were public facilities, private practice, and the home by 101 doctors (74.3%), 92 doctors (67.7%), and 58 doctors (42.7%), respectively.

Seventy‐eight doctors (66.1%) were more than 75% in favor of the use of electronic medical records. Twenty‐five doctors (21.2%) were between 50% and 75% in favor of the use of electronic medical records. Seven doctors (5.9%) of the doctors surveyed were between 25% and 50% in favor of the use of electronic medical records.

#### 3.3.2. Practices of Doctors in Relation to Telemedicine in Togo

Forty‐three doctors (31.6%) said they had never practiced telemedicine. Ninety‐three doctors (68.4%) said they practiced telemedicine. Of these, 78 doctors (57.35%) practiced teleconsultation. Respectively, 29 doctors (21.3%), 24 doctors (17.6%), 13 doctors (9.6%), and 5 doctors (3.7%) practiced tele‐expertise, medical teleassistance, medical telemonitoring, and medical regulation. Specialists and university hospital doctors were more likely to practice teleconsultation, and university hospital doctors were more likely to practice tele‐expertise, with a statistically significant difference (*p* < 0.05) (Table 4).

**Table 4:** Profile of doctors practicing teleconsultation and tele‐expertise.

	Teleconsultation			Tele‐expertise		
	*n*	%	*p*	*n*	%	*p*
Age			0.761			0.742
< 45 years old (*n* = 124)	72	58.06		27	21.77	
≥ 45 years old (*n* = 12)	6	50		2	16.67	
Professional experience			0.353			0.693
< 5 years (*n* = 64)	32	50		11	17.19	
6–10 years (*n* = 35)	23	65.71		9	25.71	
11–15 years (*n* = 21)	12	57.14		5	23.81	
> 15 years (*n* = 16)	11	68.75		4	25	
Place of work			0.99			0.664
Lomé (*n* = 87)	50	57.47		20	22.99	
Outside Lomé (*n* = 49)	28	57.14		9	18.37	
Postdoctoral training in Europe			0.276			0.275
No training (*n* = 88)	47	53.41		16	18.18	
Training (*n* = 48)	31	64.58		13	27.08	
Specialty			0.036			0.161
General practitioner (*n* = 22)	8	36.36		2	9.09	
Specialist (*n* = 114)	70	61.40		27	23.68	
Mode of practice			0.018			0.008
Hospital practicians (*n* = 90)	45	50		13	14.44	
University hospital (*n* = 46)	33	71.74		16	34.78	
Type of exercise			0.606			0.322
Private (*n* = 29)	15	51.72		4	13.79	
Public (*n* = 77)	47	61.04		20	25.97	
Private and public (*n* = 30)	16	53.33		5	16.67	

One hundred and twenty‐one doctors (89%) said they used the WhatsApp application as a means of consultation and 122 doctors (89.7%) said they used it as a means of medical prescription. One hundred and four doctors (76.5%) said they used telephone calls as a means of consultation, while 101 doctors (74.3%) said they used them as a means of prescription. Seven doctors (5.2%) and 8 doctors (5.9%) had never used remote consultation nor prescription methods, respectively.

One hundred and twenty‐two doctors (89.7%) said they had never used a connected device to take vital signs (basic clinical measurements such as temperature, heart rate, and blood pressure). Electronic medical records were not used by 118 doctors (86.8%). Sixteen doctors (11.8%) said they used an electronic medical record. Two doctors (1.5%) did not give an opinion. One hundred and twenty‐nine doctors (94.9%) expressed an interest in being trained in telemedicine.

## 4. Discussion

This study included the majority (56.4%) of doctors registered and up to date with the national order of doctors during the study period, but only 21.7% of the number of Togolese doctors (628) according to the latest statistics from the Ministry of Health [13]. This relatively small sample size reflects the low level of interest among doctors in participating in the surveys. This low participation of Togolese doctors in scientific survey studies can be largely explained by an overload of clinical work, combined with administrative responsibilities. In addition, some doctors perceive research as a secondary activity, due to insufficient training in methodology and a lack of institutional incentives. Lack of feedback on results or recognition, or the absence of concrete spin‐offs for participants, can also be obstacles. In our African countries, we therefore need to raise doctors’ awareness of the need for greater participation in scientific surveys, particularly those concerning their professions.

Our sample included a majority of specialist doctors working in the public sector. This result is similar to that reported in 2022 by Nouira and Souayeh in their study on the knowledge and attitudes of doctors towards telemedicine in Tunisia, where the majority (67.5%) were working in the public sector and 79% were specialist physicians [14]. The predominance of doctors with less than 5 years’ professional experience in our sample could be explained by the availability of young doctors and their familiarity with new information and communication technologies.

All the doctors in our study said that they had heard of telemedicine at least once. In a survey conducted among general practitioners in Gironde in 2018, Messon found that 95.3% of doctors were aware of telemedicine [15]. The study by Nouira and Souayeh in 2022 also showed that most Tunisian physicians (98.4%) have heard about telemedicine and the main sources of this information were media and colleagues [14]. It is therefore clear that, in both developed and developing countries, telemedicine is no longer a myth for doctors, thanks in particular to its popularization by the media during the COVID‐19 pandemic.

In our study, 92.7% of doctors were aware of the act of teleconsultation (a remote medical consultation between a patient and an authorized health professional, using digital communication tools). In a survey conducted among general practitioners working in multiprofessional health centers in Burgundy, France, in 2019, Berou found 82.9% of doctors who were aware of the act of teleconsultation [16]. Our sample included 61% of doctors who were aware of tele‐expertise (a remote exchange between healthcare professionals in which one physician seeks the opinion of another physician (usually a specialist) about a patient’s case, unlike Berou in France, who found that 80% of doctors were aware of tele‐expertise [16]. This discrepancy could be explained by the greater popularization of tele‐expertise in developed countries and therefore in Europe. In our study, greater awareness of tele‐expertise was found among doctors who had completed a postgraduate training course in Europe, with a statistically significant difference.

The majority of doctors, the doctors surveyed (52.9%), were aware of medical teleassistance (a real‐time remote support provided by a doctor to another health professional during a medical procedure). This is higher than that reported by Berou in France in 2019 of just 20% of doctors being aware of teleassistance [16]. Professional experience, postgraduate training in Europe, and doctors’ specialization were the factors associated with better awareness of teleassistance, with a statistically significant difference in our study. In fact, these factors are either linked to longer practice of medicine in the case of professional experience or more in‐depth practice in the case of postgraduate training in Europe and specialization. This increases doctors’ chances of being confronted with a situation in which they have to seek the help of an expert or a specialist in order to master a medical practice or in the context of training.

Medical telemonitoring, unknown to around 2/5 of the doctors surveyed in our study, is an act of telemedicine that enables a medical professional to remotely interpret the data required for a patient’s medical follow‐up and, if necessary, to make decisions relating to the patient’s care. Lastly, medical regulation (the most ignored by the Togolese doctors surveyed) is a telemedicine procedure performed over the telephone (or by means of any other telecommunication device) by a regulating doctor. Its aim is to determine and activate the most appropriate medical response for each situation as quickly as possible.

In our survey, 85.3% of doctors were aware that telemedicine is useful in daily medical practice. In a study on the successes and challenges of implementing telemedicine in the eastern provinces of Saudi Arabia, El‐Mahalli et al. found that 79% of healthcare professionals were ready to adopt telemedicine in their daily practice [17]. This high proportion found in our study could reflect the conception of telemedicine as a practice that could revolutionize and improve their medical practice. It should be noted that doctors living in the interior of the country were more aware of this usefulness, with a statistically significant difference. This can be explained by the technical and professional isolation of doctors practicing in rural areas, which unfortunately remains a reality in our developing countries. They are therefore more likely to need to seek advice from colleagues or specialists.

A third of doctors cited closer links with a hospital or contact with specialists as a benefit of carrying out telemedicine procedures. Mbemba et al. made the same observation in a study of the use and perception of telehealth by healthcare professionals in rural areas of Mali [18].

The main obstacles to the use of telemedicine cited by the doctors surveyed were organizational difficulties relating to the restructuring of the workplace, economic difficulties relating to funding and unclear means of remuneration, and the medicolegal issues [15]. As pointed out by Townsend et al. in their study on the use of e‐health by patients and healthcare professionals in British Columbia in 2015, ethical issues must be considered when developing telemedicine solutions [19]. Telemedicine raises issues such as language differences between doctors and their colleagues or patients, the legality of treatment from one country to another, questions of jurisdiction, and even health philosophy. One example is an examination of the anal area during a consultation, which is commonplace for the doctor but out of the ordinary and may even be rejected by the patient. Precise regulations are therefore needed in such cases. The security and confidentiality of information are also an important part of the medicolegal aspect. In Karachi, Pakistan, in 2020, Ashfaq et al. in their study of doctors’ knowledge and perceptions of telemedicine, found that 42.9% of doctors thought that telemedicine disrupted the doctor–patient relationship [20]. Our survey revealed that 67.7% of doctors considered telemedicine to be a necessity, compared with 74.8% in a study carried out by Ayatollahi et al. in Iran in 2015 [21]. This finding could be explained by the lack of development of telemedicine in Togo. For example, a study conducted by Meher et al. in India concerning the knowledge and perceptions of doctors and patients with regard to telemedicine showed that doctors were more interested in using telemedicine when it was available in their workplace [22].

In our study, 68.4% of doctors said they practiced telemedicine. Among the doctors surveyed, 57.4% had declared that they practiced teleconsultation. This was not the case in France in 2019, where Berou found a proportion of 26% of doctors practicing teleconsultation [16]. This difference is probably due to the fact that Berou’s study focused on general practitioners. In our series, specialist doctors and university hospital doctors were the most likely to have adopted this attitude, with a statistically significant difference.

The WhatsApp application and the telephone call were the main remote medical consultation and prescription tools used by the doctors in our study. The use of social networks such as WhatsApp has been reported by other authors in Africa [23, 24] and elsewhere in the world [1, 25]. It is not surprising to find the social network WhatsApp among the main known means of practicing telemedicine. Indeed, social networks are playing an increasingly important role in all areas of life these days. Given that these networks have no security guarantees, we can rightly question the security and efficiency of these practices. The use of the WhatsApp application to transfer personal data is subject to various risks, including piracy and theft of the phone and the information it contains. We therefore recommend that healthcare professionals use, or at least give preference to, secure private networks (medical VPN, secure intranet), locally approved telemedicine platforms or dedicated healthcare teletransmission systems for the various telemedicine acts.

Around nine‐tenths (89.7%) of doctors said that they had never used connected devices to take constant readings. These results are not similar to those found by Albarrak et al. who noted that 46.9% of doctors were familiar with telemedicine tools in 2019 [1]. This low rate of use of connected objects is thought to be due to the lack of telemedicine conferences and workshops. Studies carried out in Iran by Albarrak et al. showed that around 77% of healthcare professionals thought that continuing education sessions were essential for the development of telemedicine practice [1]. However, it should be noted that the use of connected objects is not without its drawbacks. In fact, Timmermans and Almeling have shown that modern e‐health technologies affect the doctor–patient relationship through complex social interactions leading to the commodification, commercialization, and standardization of care [26]. Doctor–patient interaction via connected objects can easily be reduced to a simple exchange of knowledge through an interface, usually a screen. This raises the question of whether the patient is not simply an image and a voice and therefore an object.

Over seven‐tenths of doctors (74.3%) listed medical imaging as the main area that should benefit from the development of telemedicine. On the other hand, Messon found that 74.8% of doctors in France identified dermatology as an area that should benefit from the development of telemedicine [15]. Medical imaging and dermatology remain the medical fields where telemedicine is easiest to implement. Indeed, these specialties present numerous cases where inspection and therefore visual information is sufficient for diagnosis. The equipment required for effective telemedicine is therefore mainly limited to a high‐definition camera and sufficient internet connectivity for data transfer.

We found that only 11.8% of doctors used an electronic medical record. This result is a direct consequence of the low level of digitization of medical practice in the country. The majority of doctors were in favor of using an electronic medical record. The use of electronic medical records is a crucial element in the implementation of telemedicine. This finding is reinforced by the study concerning the factors predicting the behavior and acceptance of telemedicine services by doctors carried out in South Korea in 2014 by Rho et al. Their study, in addition, revealed that the lack of accessibility to the patient’s medical record outside the hospital where the patient resides has a negative impact on the development of telemedicine [27].

The fact that 94.9% of respondents expressed interest in receiving training on telemedicine is consistent with international literature. In Germany, physicians reported significant training needs to ensure safe and effective practice [28], while in France, both students and practicing clinicians emphasized the need to strengthen telemedicine teaching within medical curricula [29, 30]. These findings collectively demonstrate that training is a critical determinant of adoption and acceptable practice.

The effective implementation of telemedicine in Africa is hampered by deep digital divides between urban and rural areas and the absence of national regulatory structures for telemedicine in most countries. It is therefore important to reiterate at the end of this study that bridging the digital divide and establishing national regulatory frameworks are essential prerequisites for ensuring safe, equitable, and sustainable telemedicine in Togo and other African countries.

Despite its contributions, it must be acknowledged that our study has certain limitations. Indeed, because of their workload, some doctors might not have responded to the questionnaire straight away. These answers could therefore have been given after consulting documents, which could constitute a bias in our analysis. Also, the relatively small size of our sample and the distribution of survey forms via WhatsApp platform constitute a certain limitation to our study.

## 5. Conclusion

Togolese doctors are aware and knowledgeable of telemedicine procedures such as teleconsultation, tele‐expertise, telemonitoring, and telemedicine regulation. Togolese doctors have a good perception of telemedicine, although its practice has not been formalized. The WhatsApp application was the main means of telemedicine used by doctors, despite the risks of security and confidentiality of medical data. The majority wanted telemedicine to be integrated into their daily medical practices. Improved awareness and ongoing medical training in telemedicine for doctors on the one hand, and efforts by the health authorities to digitalize medical services within a well‐defined legislative and regulatory framework on the other, will enable telemedicine to develop more effectively in Togo. Patients’ perceptions of telemedicine and, above all, the socioeconomic impact of telemedicine initiatives in communities are areas for future research in our developing countries, to be elucidated by experts in the field.

## Ethics Statement

An approval from the thesis committee of the Faculty of Health Sciences at the University of Lomé, acting as its IRB (Institutional Review Board), was obtained (No. 119/ME/21). The study did not involve the use of animals and had been performed in accordance with the Declaration of Helsinki. The physicians included in this study gave written informed consent to participate in this research.

## Consent

Please see the Ethics Statement.

## Disclosure

The manuscript has not been submitted to any other journal/site in part or in whole for consideration. It is solely submitted to this journal. All authors read and approved the final manuscript.

## Conflicts of Interest

The authors declare no conflicts of interest.

## Author Contributions

Kokou Adambounou put the idea and the design of the study. Raymondo Sêdzro Kuto, Kokou Adambounou, and Alexis Agbodjan did data collection and have contributed to the conception and design of the manuscript. Akoété Beleave Kouevidjin, Raymondo Sêdzro Kuto, and Kokou Adambounou had contributed to the conception and design of the manuscript. All authors have been involved in drafting and revising the manuscript.

## Funding

This research did not receive any specific grant from funding agencies in the public, commercial, or not‐for‐profit sectors.

## Supporting Information

The English version of the questionnaire.

## Supporting information


**Supporting Information** Additional supporting information can be found online in the Supporting Information section.

## Data Availability

The data used and/or analyzed during the current study are available from the corresponding author on reasonable request.
