# Supplementary material for: Togolese Doctors’ Awareness, Perceptions, and Practices of Telemedicine: A National Cross‐Sectional Study
Source: J Trop Med. 2026 Jan 20;2026:4658443. doi: 10.1155/jotm/4658443 (PMC12817914; doi:10.1155/jotm/4658443)
Supplement: Supplementary file 1 — Supporting Information Additional supporting information can be found online in the Supporting Information section. [file JOTM-2026-4658443-s001.pdf]

Awareness, perceptions and practices of telemedicine by Togolese doctors

A- SOCIODEMOGRAPHIC CHARACTERISTICS OF DOCTORS

1- Gender : Male ☐ Female. ☐

2- Age : < 25 years ☐; 26 years-35 years ☐; 36-45 years ☐; 46-55 years ☐ ; 56 - 65 years old ☐; > 65 years old ☐

3-Qualification of the doctor : University hospital ☐; hospital practitioner ☐; Other ☐

4- Specialty of the doctor : Anatomo-pathology ☐; Biology ☐; cardiology ☐; surgery ☐; dermatology ☐; HGE ☐; ENT ☐; Ophthalmology ☐; neurology ☐ ;gynecology ☐; pulmonology ☐; psychiatry ☐; radiology ☐; rheumatology ☐; urology ☐; oncology ☐; nephrology ☐; internal medicine ☐; general medicine ☐; other ☐

5-Professional experience (years): 0-5 ☐; 6-10 ☐; 11-15 ☐; > to 15 ☐

6- Country of training in general medicine: Togo ☐; other ☐

7- Country of specialization : Togo ☐; other ☐

8- Have you had to complete a post-doctoral or specialist internship in Europe:

No ☐ / Yes ☐

8a- If yes, specify the country: …………….

9- Current sector of practice: Private ☐/ Public ☐/ Both ☐

10-City of practice: Lomé ☐/ Other ☐

11 - What is the speed of the internet connection in your place of work ?

- Very fast☐
- Good☐
- acceptable☐
- mediocre☐
- no internet connection☐

B- DOCTORS' AWARANESS ABOUT TELEMEDICINE IN TOGO

12- Have you ever heard of telemedicine?

No☐ / Yes☐

13-If yes, by what means?

Media ☐/ Faculty of Medicine ☐/ Medical Journal ☐/ Conference ☐/ from a colleague ☐/ Other☐

14- Among the following telemedicine acts, which one(s) are you aware?

-Teleconsultation☐

-Tele-expertise☐

-Medical telemonitoring☐

-Medical teleassistance☐

- Medical regulation☐

-None☐

15- What solutions/applications/means of exercising remotely are you aware?

…………………………………………………………………………………………

16- Are you aware that telemedicine is useful in daily medical practice ?

No ☐/ Yes☐

16a- Are you aware of any training methods for learning how to practice telemedicine?

No ☐/ Yes☐

16b-If yes: which one(s)? e-learning ☐/ videoconferencing ☐/ DU ☐/ Telemedicine Day☐ - Other ☐………………

17- Have you ever heard of a former or current telemedicine activity or project in Togo?

No ☐/ Yes☐

18- For you, what will be the main benefit in your practice of carrying out acts of Telemedicine?

- Contact with a hospital structure (contact with specialists) ☐
- Benefits for patients (time saving/less travel) ☐
- Help combat medical desertification in rural areas☐
- Distance learning☐
- Tele-expertise☐
- medical telemonitoring☐
- Other ☐…………………

19- What do you think is the main obstacle(s) to being able to use the Telemedicine ?

- Organizational brake (restructuring to implement such a technique) ☐
- Economic brake (development financing, remuneration still unclear) ☐
- Lack of motivation☐
- Lack of time (fear of wasting time with such a practice) ☐
- Lack of credibility with patients☐
- Incomplete review☐
- Forensic aspect ☐
- None☐
- Other ☐……….. ……. .

20- Is the patient's consent obtained for the collection and sharing of his/her information with other colleagues?

No ☐/ Yes☐

C- PERCEPTIONS AND PRACTICES OF DOCTORS IN RELATION TO TELEMEDICINE IN TOGO

21- For you, telemedicine is:

- science fiction ☐
- a necessity ☐
- a matter for specialists ☐
- a hope or the future of medicine ☐
- not concerned ☐

22- Among the following telemedicine acts, which one(s) do you practice?

-Teleconsultation ☐

-Tele-expertise ☐

-Medical telemonitoring ☐

-Medical teleassistance ☐

- Medical regulation ☐

-None ☐

23- Have you ever consulted a patient by one of these virtual paths ? :

Phone call ☐ / WhatsApp ☐ / Facebook ☐ / never ☐ / Other ☐

24-Have you ever prescribed a prescription to a patient through one or more of these virtual channels?

Phone call ☐ / WhatsApp ☐ / Facebook ☐ / Never ☐ / Other ☐

25-Have you ever received a paraclinical result from a patient through one or more of these virtual channels?

Phone call ☐ / WhatsApp ☐ / Facebook ☐ / Never ☐ / Other ☐

26-Have you ever received a paraclinical result from a colleague through one or more of these virtual channels?

Phone call ☐ / WhatsApp ☐ / Facebook ☐ / Never ☐ / Other ☐

27-Have you ever taken patient vital signs using one or more of these connected objects?

Smart watch ☐/ smart bracelet ☐/ smart scale ☐/ never☐

28- Would you like to train in this practice?

No ☐/ Yes☐

29- In which areas would it be interesting to develop telemedicine procedures?

- General medicine☐
- Medical imaging☐
- Dermatology☐
- Pediatrics☐

-Gynecology☐

- Cardiology☐
- HGE☐
- Internal medicine☐
- Neurology☐
- Psychiatry☐

-Other ☐………………………

30- In which place(s) would you agree to practice telemedicine acts?

- House☐
- Liberal cabinet☐
- public structure☐
- Other ☐…………………

31- Do you use a computerized patient record?

No ☐/ Yes☐

32- If not, to what extent (%) would you be in favor of using computerized patient records?

-< 25%☐

-between 25 and 50% ☐

-between 50 and 75% ☐

-> at 75% ☐
